# 3D Data Denoising via Nonlocal Means Filter by Using Parallel GPU Strategies

**DOI:** 10.1155/2014/523862

**Published:** 2014-06-16

**Authors:** Salvatore Cuomo, Pasquale De Michele, Francesco Piccialli

**Affiliations:** Department of Mathematics and Applications “R. Caccioppoli”, University of Naples “Federico II”, Via Cintia, 80126 Napoli, Italy

## Abstract

Nonlocal Means (NLM) algorithm is widely considered as a state-of-the-art denoising filter in many research fields. Its high computational complexity leads researchers to the development of parallel programming approaches and the use of massively parallel architectures such as the GPUs. In the recent years, the GPU devices had led to achieving reasonable running times by filtering, slice-by-slice, and 3D datasets with a 2D NLM algorithm. In our approach we design and implement a fully 3D NonLocal Means parallel approach, adopting different algorithm mapping strategies on GPU architecture and multi-GPU framework, in order to demonstrate its high applicability and scalability. The experimental results we obtained encourage the usability of our approach in a large spectrum of applicative scenarios such as magnetic resonance imaging (MRI) or video sequence denoising.

## 1. Introduction

Image denoising represents one of the most common tasks of image processing. Several techniques have been developed in the last decades to face the problem of removing noise from images, still preserving the small structures from an excessive blurring (see [1] for a review). All those schemes share the belief that an improved value of a given image point can be expressed as a function of the image itself.

One of the most performing and robust denoising approaches is the Nonlocal Means (NLM) filter, introduced in 2005 by Buades et al. [2]. Since its first appearance, the family of NLM algorithm and implementation variants has enormously grown (just to mention some of the most relevant improvements, see [3–10]); nevertheless, all of them assume that the restoring function for a given point is a mean of all the image values, largely weighted according to the radiometric similarity between values and only weakly tied to a spatial proximity criterion.

The result is a general-purpose denoising scheme, whose performances are widely accepted to be better with respect to the previous state-of-the-art algorithms, such as the total variation, the wavelet thresholding, or the anisotropic filtering [11]. In particular, it has been shown that NLM filter guarantees the homogeneity of flat zones, preserves edges and fine structures, and transforms white noise into white noise, thus avoiding the introduction of artifacts and spurious correlated signal [1]. Unsurprisingly, the NLM algorithm is simple but computationally very heavy, because it requires, for denoising of each pixel, a high number of elementary operations, and even some fast versions of the scheme are quite demanding on 2D images and almost daunting on 3D datasets. The huge amount of computational demand has been recently addressed by using accelerated hardware [12, 13], the graphic processor units (GPUs) in particular.

The GPUs are massively parallel architectures that efficiently work with impressive performance improvements but at the same time require a deep understanding of the underlying architecture and* ad hoc* thread based algorithms. Obtaining reasonable performance in terms of execution times of the NLM algorithm allows its implementation in a large spectrum of applicative scenarios such as magnetic resonance imaging (MRI) or video sequence denoising where, especially in the latter one, the input datasets can reach a considerable size. Moreover, in 3D datasets, for example, in the context of the magnetic resonance imaging (MRI), the use of fully 3D filters is more appropriate than a 2D-based slice-by-slice filtering approach to exploit all the information contained in the image. To the best of our knowledge, although there are several 2D GPU-based NLM versions [14–16], the 3D version of NLM filter has been poorly investigated in terms of both implementation and performance on GPUs.

In this paper we design and implement a fully 3D Nonlocal Means parallel approach based on Compute Unified Device Architecture (CUDA) [16] that relies on the graphic processor unit (GPU) architecture. Moreover, we adopt a simple but effective strategy to avoid the GPU memory bound issue, due to oversizing 3D datasets, for example, in a video sequence denoising scenario. We report and analyze the performance of our implementation for several 3D datasets. The parallelization strategy of the filter via GPUs gives clinically-feasible MRI denoising execution times with a significant increase in speed.

The plan of the paper is as follows. In Section 2 we briefly describe the NLM algorithm. To follow, in Section 3 we provide the implementation details. Moreover, in Section 4 we report a brief review about the related works and in Section 5 we present and discuss the results of our work. Finally, in Section 6 we draw conclusions and outline the future work.

## 2. Theory

### 2.1. General Description

An *N*-D image *X* can be considered as a real function *X* : *R* ^*N*^ → *R* with a bounded support Ω ⊂ *R* ^*N*^. The NLM filter [2] is a class of endomorphisms of the image space, identified by 2 parameters (*a* and *h*), that acts as follows:

(1) $$\begin{array}{c} \left[\text{NL}{\text{M}}_{a,h}\left(X\right)\right]\left(x\right)=Y\left(x\right)=\frac{\int_{\Omega }e^{-d_{a}^{2}(x,y)/h^{2}}X\left(y\right)dy}{\int_{\Omega }e^{-d_{a}^{2}(x,y)/h^{2}}dy}, \end{array}$$

where

(2) $$\begin{array}{c} d_{a}^{2}\left(x,y\right)\equiv \int_{R^{N}}{\left|X\left(x+t\right)-X\left(y+t\right)\right|}^{2}\cdot \frac{e^{-{||t||}^{2}/2a^{2}}}{{\left(2\pi \right)}^{n/2}\cdot a}dt. \end{array}$$

The intensity of a given point of the new image is a mean of the intensities of the original image, according to a weight function that disregards any explicit criterion of spatial proximity and only considers a measure (ruled by *h*) of self-similarity between windows of radius *a* centered on each point (radiometric proximity).

If the image is defined on a discrete, regular grid {**x**_*i*_∣1 ≤ *i* ≤ ∏_*l*=1_^*N*^ *L*_*l*_},

(3) $$\begin{array}{c} X\left(x\right)=\underset{i}{\sum }X_{i}\delta \left(x-x_{i}\right). \end{array}$$

From (1)-(2) it follows that the filtered dataset is given by

(4) $$\begin{array}{c} Y_{i}=\frac{\sum_{j}e^{-d_{a}^{2}(x_{i},x_{j})/h^{2}}X_{j}}{\sum_{j}e^{-d_{a}^{2}(x_{i},x_{j})/h^{2}}}, \end{array}$$

(5) $$\begin{array}{c} d_{a}^{2}\left(x_{i},x_{j}\right)=\underset{k}{\sum }{\left|X\left(x_{i}+\Delta_{k}\right)-X\left(x_{j}+\Delta_{k}\right)\right|}^{2}\cdot \frac{e^{-{||\Delta_{k}||}^{2}/2a^{2}}}{{\left(2\pi \right)}^{n/2}\cdot a}. \end{array}$$

Moreover, from (4)-(5) it follows that the complexity of the filter is *O*(∏_*l*=1_^*N*^ *L*_*l*_^3^).

### 2.2. Actual Algorithm

Both computational issues and the convenience to introduce a geometric proximity criterion in addition to the pure radiometric distance measure led to a change in the original version of the NLM filter [6].

Therefore, given a search radius *M*, for each voxel *i* located at **x**_*i*_ we define a search box *V*_*i*_ as

(6) $$\begin{array}{c} V_{i}\equiv \left\{x_{j}\in \Omega \;|\;{||x_{j}-x_{i}||}_{\infty }<M\right\}. \end{array}$$

The search box associated with the *i*th voxel defines the ensemble of voxels whose intensities will be available in the following for restoring (denoising) of the intensity *X*(**x**_*i*_), thus reducing the search freedom of (4) (in that case, *V*_*i*_ ≡ Ω).

Analogously, given a similarity radius *d*, for each voxel **x**_*j*_ within a given search box *V*_*i*_, we can define a similarity box

(7) $$\begin{array}{c} {\;}_{j}B_{i}\equiv \left\{x_{k}\in \Omega \;|\;{||x_{k}-x_{j}||}_{\infty }<d\right\}. \end{array}$$

In this case, *d* plays the role of *a* in (4), provided that the original smooth Gaussian kernel is replaced by a binary cut-off.

Finally, the denoised image is

(8) Yi=∑xj∈Viexp⁡[−||Bij−iBi||22/h2]Xj∑xj∈Viexp⁡[−||Bij−iBi||22/h2],

where it results that the algorithm complexity is *O*(|*V*_*i*_ | ·|_*j*_ *B*_*i*_ | ·∏_*l*=1_^*N*^ *L*_*l*_).

The filter strength, which is determined by *h*, can be tuned to obtain an optimized denoising, independently from the search radius *M* and the standard deviation of noise *σ*, where *β* ~ 1 is an adimensional constant to be tuned:

(9) $$\begin{array}{c} h^{2}=2\beta \sigma^{2}\left|V_{i}\right|. \end{array}$$

In Algorithm 1 the pseudocode of the NLM filter is showed.

## 3. Parallel GPU Implementation

General-purpose computing on graphics processing units (GPGPU) is the use of GPUs to perform highly parallelizable computations that would normally be handled by CPU devices. Programming with GPUs requires both a deep understanding of the underlying computing architecture and a massive rethinking of existing CPU based algorithms. The basic idea behind GPGPU is handling the sequential code by CPU and processing parallel code on GPU, still having CPU be the coordinator of the data processing. Additionally, the use of multiple GPU in one system, or large numbers of graphics chips, further parallelizes the already parallel nature of graphics processing.

**Figure 1:**
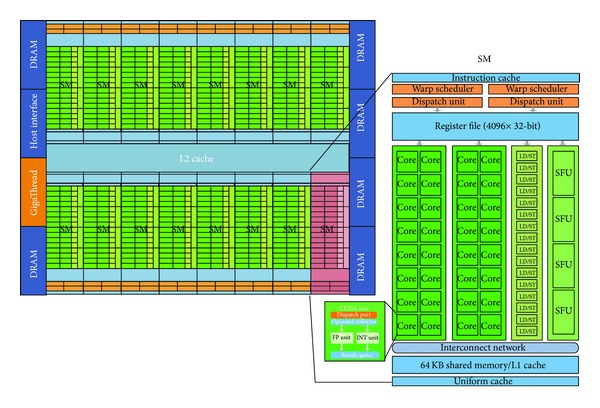
View of a NVIDIA FERMI GPU architecture.

We implement the 3D NLM filter on the NVIDIA parallel computing architecture, which consists in a set of cores, or scalar processors (SPs), performing simple mathematical operations. SPs are organized into a streaming multiprocessor (SM), which have a shared memory, a shared L1-cache, and several other units. A SM executes one or more thread blocks, while CUDA cores and the other units execute thread instructions; in particular, each SM executes threads in groups of 32, called warps [17]. In Figure 1 we show the NVIDIA Fermi architecture, where each SM (vertical rectangular strip) has scheduler and dispatch units (orange portion), execution units (green portion), and a configurable memory of 64 KB (light blue portions), which consists of a register file, an internal shared memory, and an L1 cache. This memory is configurable in 16 KB (or 48 KB) for shared memory and 48 KB (or 16 KB) for L1 cache. Moreover there are 16 load/store (LD/ST) units per SM and 4 special function units (SFUs) (nuances of green portions) available to handle transcendental and other special operations such as  sin,  cos,  exp, and  rcp (reciprocal).

### 3.1. GPU Thread Strategy Mapping

As shown in Algorithm 1, the computational factor that influences the overall performance of this algorithm is represented by a nested iteration structure (see the statement at line 1). A basic serial implementation is presented in Algorithm 2.

As we can observe, the nested* for loops* represent the main bottleneck in the algorithm computation; according to this, the first step of our parallelization strategy is to* unroll* the outermost* for loops* that, in the serial version of the algorithm, perform the scanning of the 3D image.

**Figure 2:**
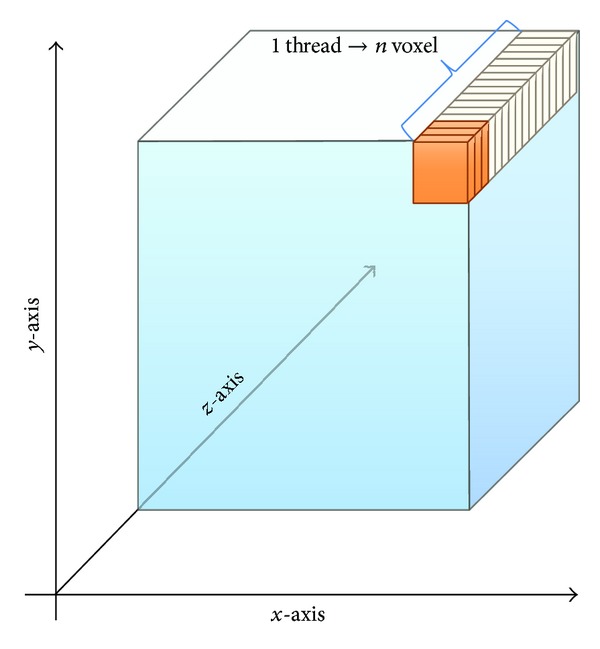
Graphic representation of the partial unrolling strategy.

With the aim of achieving the best performance of the algorithm and to optimize the usage of this massively parallel architecture, we perform two different* unrolling* strategies. In the first one, illustrated in Figure 2, we unroll the* for loops* that scan on *x*-axis and *y*-axis, leaving the* for loop* on *z*-axis (partial unrolling); in this way the GPU model proposes that each thread is in charge of performing the denoising process of all the voxels in its *z* direction (see Algorithm 3).

**Figure 3:**
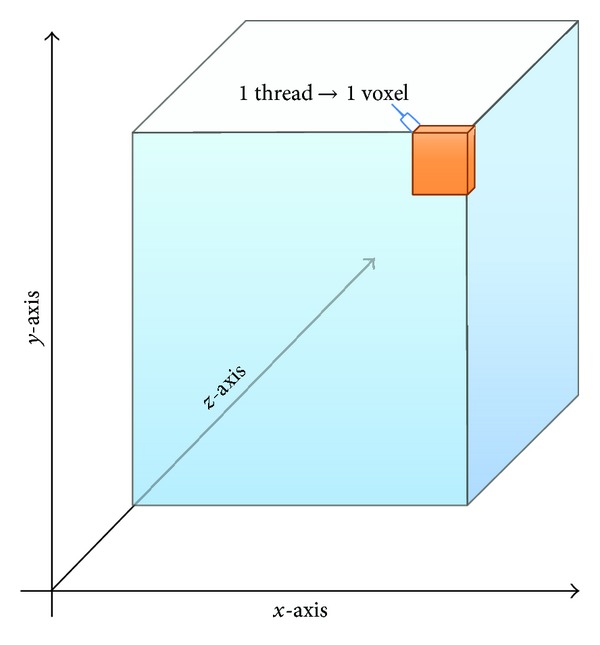
Graphic representation of the full unrolling strategy.

In the second strategy, illustrated in Figure 3, we unroll all the* for loops* that scan the 3D images along the directions *x*, *y*, and *z* (full unrolling); in this way the GPU model proposes that each thread is in charge of performing the denoising process of the single voxel associated to it (see Algorithm 4).

These two strategies require different kernel grid configurations, in order to take full advantage of the GPU architecture; while the first strategy aims at creating a smaller number of threads with a longer computing time, the second one aims to create a larger grid and therefore a greater number of threads, which, however, have a shorter computing time.

We clearly observe that these implementations are bounded by the GPU global memory size. Since, for a fixed dataset Ω, we store two objects to manage the overall data (the first one for the input dataset and the second one for the denoised one), the maximum size of available memory is *K* = *M*/2, where *M* is the GPU global memory size in byte. For datasets that exceed *K* we implement an* ad hoc* function that tunes and splits the data before starting the denoising. In particular, this function follows the following.

1. Compute the available memory *K* and the size of the dataset Ω.
2. Compute the size of each subdataset Ω_*i*_ (with *i* = 1,…, *N*, where *N* is the number of subdatasets) that depends on the search (|*V*_*i*_|) and similarity (|_*j*_ *B*_*i*_|) window cardinalities.
3. Split the dataset Ω into *N* subdatasets Ω_*i*_.

**Figure 4:**
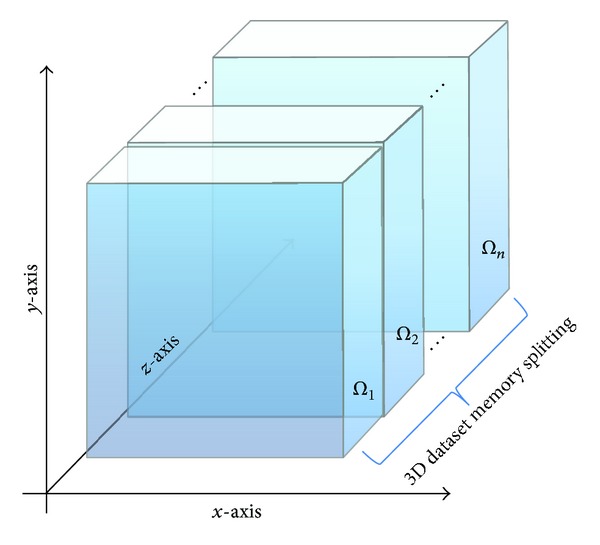
Graphic representation of the split.

The function, in a first phase, splits the input dataset along the *z*-axis; then the 3D NLM GPU kernel is executed for each Ω_*i*_, exactly *N* times, and finally, at the end of the denoising process, it performs a* gathering* phase, in order to reassemble the entire dataset collecting the different denoised parts from each execution of the kernel function. It should be noted that the size of each subdataset Ω_*i*_ is computed taking into account an overlapping zone between two adjacent subdatasets, to adapt the NLM algorithm 3D on suitable search and similarity windows. In Figure 4 the graphic representation of the split along the *z*-axis is showed, where, to simplify the discussion, we do not consider the overlapped portions.

**Figure 5:**
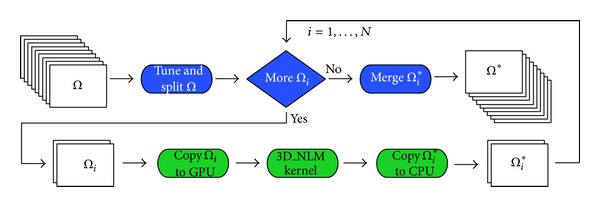
3D NLM work flow.

For a better understanding of the entire process and its different steps that compose the 3D NLM GPU algorithm, we show in Figure 5 its work flow, where the blue colored charts represent the CPU workload and the green colored charts represent the GPU one. The input dataset Ω is splitted in *N* subdatasets Ω_*i*_, by the “tune and split” function. One at a time, each Ω_*i*_ is copied from the CPU (host) to the GPU (device), then it is processed by the 3D NLM kernel and the resulting denoised subdataset Ω_i_* is copied from the GPU to the CPU. Finally, all the Ω_i_* are merged in order to obtain the denoised dataset Ω*.

### 3.2. Multi-GPU Implementations

To exploit even larger degrees of parallelism in our 3D dataset denoising, we deploy a new version of the 3D NLM algorithm that is compatible with multi-GPU architectures. The multiple GPUs avoid many of the limitations of single GPU utilization (e.g., on-chip memory resources) by exploiting the combined resources of multiple devices. Dividing the algorithm across multiple GPUs requires partitioning the computational space and allowing each GPU to operate on a subsection of the data. Although, during all our experiments, we consider only configurations with one or two GPU, the framework is fully compatible to exploit the use of more than two GPUs.

It is important to underline that the input dataset will be partitioned among the global memories of the GPUs by means of the* ad hoc* function for tuning and splitting, above described. Also in this case, we split the input dataset along the *z*-axis, thereby distributing the slices among the available GPUs; then we allocate on each GPU global memory only the required space to store the subset of the input data. At the end of the denoising process, we perform a* gathering* phase, in order to reassemble the entire dataset collecting the different denoised parts from each GPU. As we will show in the next section, the transfer time between GPU global memory and the host RAM memory minimally affects the total computing time of the algorithm.

**Figure 6:**
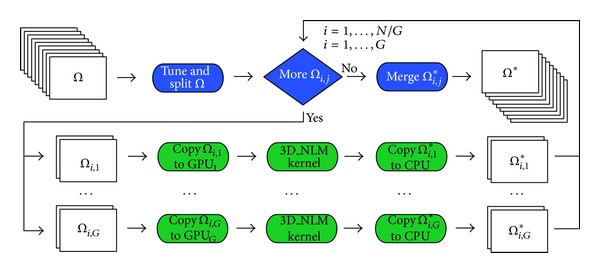
3D NLM multi-GPU work flow.

In Figure 6 we report a simple flowchart that summarizes our multi-GPU strategy. Let *G* be the number of available GPU units and *N* the number of subdatasets Ω_*i*,*j*_ (where *i* = 1,…, *N*/*G* and *j* = 1,…, *G*). If the sizes of the input and output datasets, respectively, Ω and Ω*, are bounded by the total GPU global memory size, then *N* is a multiple of *G*, with *N* > *G*; otherwise *N* = *G*. As we can observe, the input dataset Ω is splitted into *N* subdatasets Ω_*i*,*j*_, by the “tune and split” function. At this point, the Ω_*i*,*j*_ are copied from the CPU to the available GPU units that, independently of one another, perform the denoising of the assigned Ω_*i*,*j*_. Then the resulting denoised subdataset Ω_*i*,j_* is copied from the specific GPU unit to the CPU. Finally, all the Ω_*i*,j_* are merged in order to obtain the denoised dataset Ω*.

Exploiting the multi-GPU support will lead to a drastic reduction of the execution times as we will detail in Section 5.1. This kind of approach allows us to take advantage of a second type of parallelism; indeed we observe that the denoising of a single slice along the *z*-axis is independent from the denoising of the other slices along the same axis; therefore it is possible to split the dataset into subsets of contiguous slices that can be processed in parallel by several GPUs.

The basic idea consists in the identification of the number of GPU devices on the architecture; this information can be returned by means of a CUDA library function and stored in the variable n_gpus. Then, the third dimension of the image is “splitted” between the available GPUs. In Algorithm 5, we report a scratch of the multi-GPU implementation with a partial unrolling strategy. For the multi-GPU algorithm with full unrolling strategy, the implementation is in a similar way.

**Figure 7:**
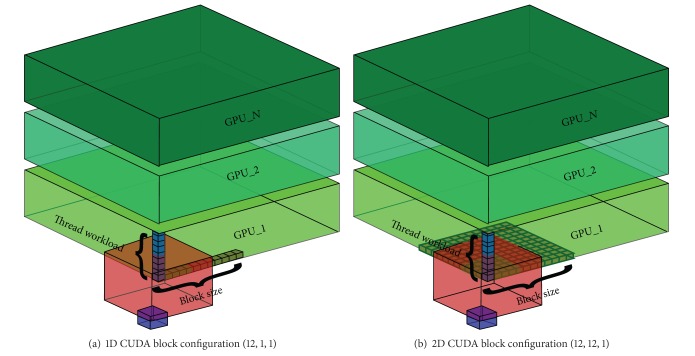
Schematic representation of thread organization inside a single GPU and between GPUs. Thread workload highlights the voxels that will be processed by a single thread. Moreover, threads can be organized in strips or tiles of the specified Block size. The red cube depicts the search window and the smaller blue one depicts the similarity window.

As data access times in memory can vary depending on the kernel configuration, we focus our attention on testing several configurations, both mono- and bidimensional, for the thread block size in which each slice is divided; therefore, each thread processes sequentially the voxels along the third dimension. The workload is divided along the third dimension for multi-GPU configurations. Inside each GPU the workload is divided along the first and second dimensions, in strips (monodimensional configurations) and tiles (bidimensional configurations) of threads. Strips or tiles are allowed to cover entirely or only partially the slice grid. In Figure 7 we report a schematic representation of the data processing subdivision.

Moreover, we also test the performance impact of L1-cache usage, by using the binary L1-prefer setting, which allows choosing between two possible configurations: 48 KB of shared memory and 16 KB of L1-cache (no L1-prefer), or 16 KB of shared memory and 48 KB of L1-cache (L1-prefer).

The computing environment we used is equipped with 2 Intel Xeon CPU E5620 (2.4 GHz) and an NVIDIA TESLA S2050 card, based on the FERMI GPU. This device consists of 4 GPGPU units, each of which with 3 GB of RAM memory and 448 processing cores working at 1.15 GHz. Moreover, the middleware framework has OS Linux 2.6.18-194.el5, CUDA release 3.2, and C compiler gcc v.4.1.2. The numerical code is implemented by using the single precision arithmetic. The CPU system is equipped with an INTEL core i5-2500S (Sandy Bridge) at 2.7–3.7 GHz.

## 4. Related Work

General-purpose computing on graphics processing units (often termed GPGPU or GPU computing) supports a broad range of scientific and engineering applications, including physical simulation, signal and image processing, database management, and data mining [18–22].

Recently, a number of authors have implemented the 2D Nonlocal Means algorithm on a GPU; in this section, we will describe representative ones. In [23] a locally constant weight assumption is used in the GPU implementation to speed up the basic algorithm. In [15], a GPU extension of the Nonlocal Means algorithm is proposed to denoise ultrasound images. In this approach, the maximum patch size is limited by the amount of shared memory of the GPU. A technique to incorporate both temporal information and color information into the Nonlocal Means algorithm, in order to restore video sequences, is described in [16]. From a parallel computational point of view, they did not report in depth analysis of the accelerated algorithm; moreover the algorithm they consider is basically a bidimensional implementation, that although it is computationally less expensive, is not able to reach the same denoising quality results of the 3D version we consider and implement in this paper.

## 5. Results and Discussions

In this section we want to investigate and discuss the performance and the scalability of the proposed GPU algorithm, taking into account also the splitting strategy. To better point out the effective application of this implementation, we report, in Section 5.1, a magnetic resonance imaging case of study.

### 5.1. A Case of Study: MRI Consistency

**Figure 8:**
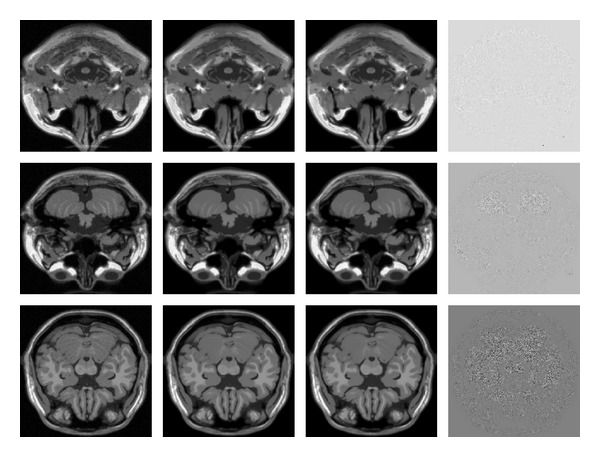
From left to right the frames show a slice of the original dataset, the CPU restored image, the GPU restored image, and the difference between CPU and GPU filtered images (enhanced by a scaling factor of 10^6^). From top to bottom, there are initial, central. and final slices.

As demonstrated in these research articles [24, 25], magnetic resonance imaging can take great advantage of the Nonlocal Means filter, since the MRI images always suffer of Rician noise. Therefore, in order to produce a strictly equivalent GPU implementation of the sequential CPU NLM algorithm, we check the implementation consistency by comparing voxel-by-voxel the images obtained by CPU and GPU denoising. In Figure 8 we show the 3D NLM filtering results, where the overall simulations are carried out with a 3D brain MRI dataset (http://www.bic.mni.mcgill.ca/brainweb) of 217 × 181 × 181. The difference between the GPU and CPU restored images falls within machine precision order of magnitude, which are likely to be due to the arithmetic logic unit precision. Since the differences are negligible (six orders of magnitude smaller than signal), they do not impair visual inspection.

### 5.2. Performance

To demonstrate the advantages and benefits of using our parallelization strategy we report several performance experiments. From a memory usage optimization point of view, we focus our attention on the impact of the cache size on the overall execution time and perform several test runs by varying L1-prefer modality; promising results are shown in Table 1, both for the partial unrolling algorithm and the full unrolling algorithm. Clearly, L1-prefer choice gives a benefit on lager dataset, with a performance improvement that ranges from fraction of percent in the smallest dataset to some 5% in the largest ones. From the other side, as we can observe in Table 1, forcing the L1-prefer setting gives a performance that benefits only the partial unrolling strategy. These results suggest that the L1 miss rate, still with 16 KB, is low enough to have high performance processing even with old generation cards having small amount of cache.

As we mentioned in the previous section, the kernel grid configuration plays a key role if we want to optimize the executions of the GPU NLM filter. The strip or tile thread division influences the filter performance in terms of computing time, due to the different type of data access. Experimental results we carry out prove that an optimal configuration is given by the strip subdivision and in Table 1, both for the partial unrolling algorithm and the full unrolling algorithm; we report the running times of (128,1, 1) configuration on 2 GPUs. Adopting these different kinds of grid organization, Tables 2 and 3 (both for the partial unrolling algorithm) and Tables 4 and 5 (both for the full unrolling algorithm) summarize a detailed comparison between running times of CPU, single GPU, and multi-GPU implementation of 3D NLM filter with various thread block size. Speed-up values suggest that the bigger the dataset to be filtered, the better the scalability of the implementation. In particular, for datasets size typical of MRI clinical practice, the scalability of the proposed implementation is close to be ideal. Moreover, the optimal thread configuration seems to be strips of thread between 128 and 256 elements. This result is strongly consistent with NVIDIA guidelines [26] about optimal thread block size. Finally, the obtained results suggest that a strip configuration should be preferred to tile one of the same size, because of sequential memory access.

**Figure 9:**
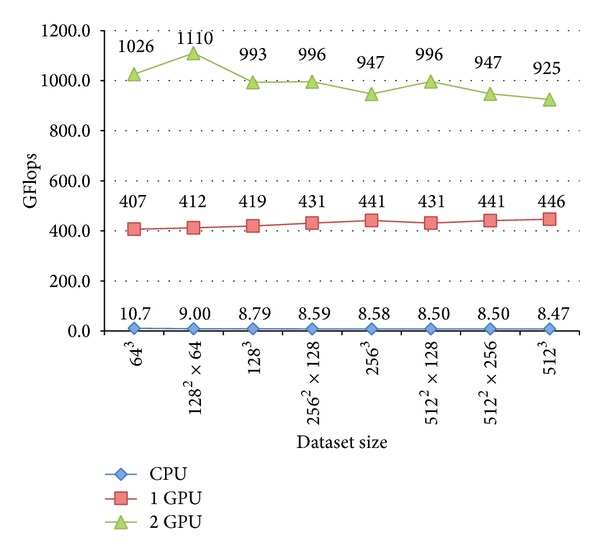
Outline of the CPU (blue), 1 GPU (red), and 2 GPU (green) GFlops values for the full unrolling algorithm with different dataset sizes and thread block configuration (128,1, 1).

In Figure 9 we outline the CPU (blue curve), 1 GPU (red curve), and 2 GPU (green curve) GFlops for the full unrolling algorithm with variable dataset sizes and thread block configuration (128,1, 1) (see Tables 4 and 5), since the performance calculation of an algorithm through GFLOPs is very indicative in areas where the floating point calculation is the predominant feature of the calculation itself. It should be remarked that we are able to exploit up to 55% of single precision floating point peak performance of the used GPU.

Another factor that can strongly influence the performance trend of the algorithm is the size of the search and similarity windows. Hence we investigate the behavior of the full unrolling algorithm, with a 3D dataset of normally distributed random numbers (size = 512 × 512 × 128), against the search (|*V*_*i*_|) and similarity (|_*j*_ *B*_*i*_|) window cardinalities. In our experiments we consider the search and similarity window size to be larger than the common dimension in the real scenarios. Observing Tables 6 and 7, respectively, for executions on 1 GPU unit and 2 GPU units, we note a high and almost constant speed-up among the various experiments, which makes feasible large window filter testing in a reasonable time.

At the end of this section we draw some practical remarks. To have the best hardware performance and results closer to NVIDIA guidelines [26], configuration tricks are needed. It is strongly recommended to use the L1-prefer feature in the partial unrolling algorithm. This fact is related to the principle locality of the threads for data accesses. Moreover, in overall experiments, we have observed that the optimal thread configuration seems to be strips of thread between 128 and 256 elements.

### 5.3. GPU NLM Video Denoising

The performance results showed in the previous section encourage the application of this denoising filter to a number of different scenarios, formerly far away from being able to be taken into account, due to the filter high computational demand on large datasets. Denoising video sequences represent one, where the increase of resolution and time of a video sequence would lead to denoising times that do not take into account adopting a serial version of the filter. According to this, we perform several performance tests on different video in resolution and fps, in order to demonstrate the scalability of our splitting approach on huge input datasets. Selecting the most commonly used video formats, PAL (720 × 576), HD (1080 × 720), and FULL-HD (1920 × 1080), we report the execution times for the 3D NLM GPU kernel with the partial unrolling strategy, in Table 8, and with the full unrolling one in Table 9.

**Figure 10:**
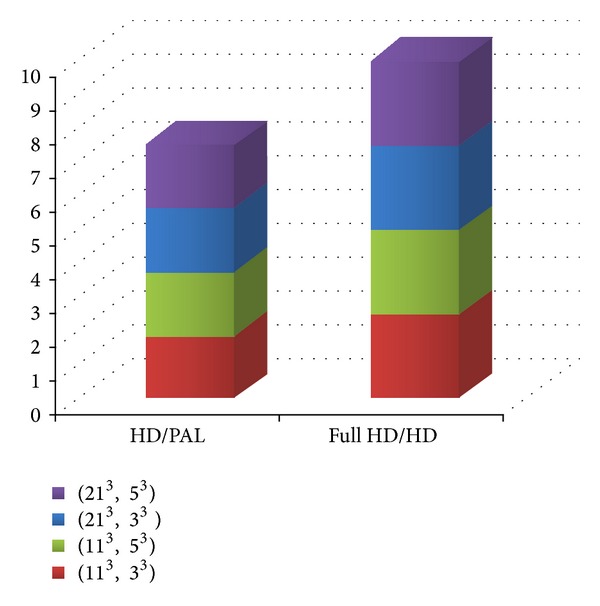
Scalability of the full unrolling approach, fixed the third dimension (fps).

Investigating more in depth the scalability of our full unrolling approach, in Figure 10, we fixed the third dimension (fps) and tuned the first two dimensions (see the related Tables 8 and 9). In this case, the algorithm well scales with each research windows configuration. Moreover, in this chart we correlate the ratio between the HD format execution times with respect to the PAL one and the FULL-HD format with respect to the HD one; each color represents a research window configuration of the NLM algorithm. In both histograms, the colored cubes have approximately the same dimension between them, which indicates that the ratio between the execution time remains almost constant.

**Figure 11:**
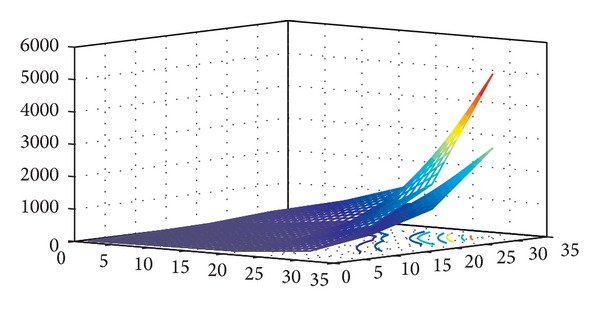
Scalability of the full unrolling approach, fixed the PAL format, and tuning the fps along the *z*-axis.

In Figure 11, we fixed the PAL format and tuned the fps along the third dimension, in order to investigate the scalability of the full unrolling algorithm, for a selected research window of the NLM algorithm.

On the *x*-axis we have four PAL videos differing in fps (50, 100, 200, and 500), on *y*-axis we have four research window sizes (10 = (11^3^, 3^3^), 20 = (11^3^, 5^3^), 30 = (21^3^, 3^3^), and 40 = (21^3^, 5^3^)), and on *z*-axis we have the execution times. The below surface represents the ideal execution times that would be obtained if the algorithm perfectly scales, growing the third video dimension (fps). The top surface instead represents the execution times actually obtained by the algorithm. As we can observe, the two surfaces deviate from each other and this phenomenon grew considerably with the increase of the values along the third dimension. Accordingly, it was necessary to modify the algorithm to increase scalability, adopting the splitting strategy described in the previous section. In detail, splitting a video sequence, in subsequences at 50 fps, we obtain execution times proportional to increasing fps, while augmenting the overall fps number. As example, considering a video sequence of 100 fps, the execution time of the algorithm will be approximately twice with respect to a sequence at 50 fps (both the same format; see Table 10); the time necessary to transfer the data from the CPU to the GPU and conversely is negligible.

**Figure 12:**
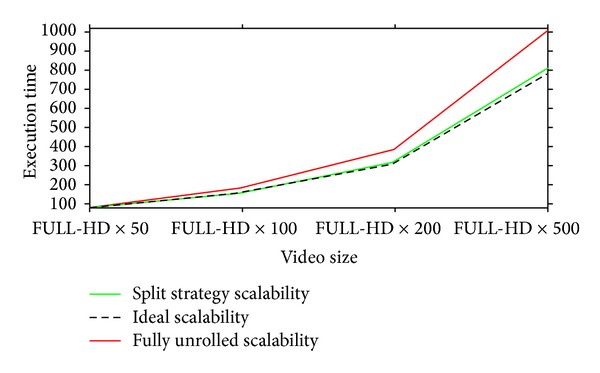
Scalability comparison between the fully unrolled strategy, the splitting strategy, and the ideal, for the FULL-HD video format.

Finally, in Figure 12, we report a very encouraging result in terms of scalability; on *x*-axis we have 4 FULL-HD videos differing in fps (50, 100, 200, and 500) and on *y*-axis we have our GPU NLM denoising execution times. In the chart, the dashed line represent the ideal scalability, the red line the scalability obtained by using the fully unrolled strategy, and the green line the scalability by using the splitting strategy. As we can observe, our splitting strategy applied on the GPU version of NLM algorithm presents a scalability very close to the ideal one.

## 6. Conclusions

NLM filter is a state-of-the-art denoising algorithm. However, the huge amount of computational load prevents the large-scale diffusion of most common implementations of the algorithm, in particular for 3D datasets of great sizes.

In this paper we design and implement a fully 3D Nonlocal Means parallel approach, adopting different algorithm mapping strategies on a GPU and multi-GPU architecture in order to demonstrate its high scalability and consequent applicability. We focus our attention on designing and testing several kernel configurations that rely on two different threads usage schemes: bidimensional and tridimensional CUDA kernels adopting a multi-GPU framework. In this way, we argue to identify a set of optimal settings that guarantee high performance results in terms of execution time and scalability of this approach, for a wide spectrum of application scenarios. Moreover, we propose two different cases of studies; the first simulates an MRI scenario, where the Nonlocal Means algorithm is widely applied to remove the Rician noise in this kind of medical images; the second simulates a video sequence that represents a suitable example of huge 3D dataset.

The obtained running times and the scalability demonstrate that, for most common 3D dataset sizes, thanks to our parallelizing approaches, we are able to drastically reduce the inapplicability of the Nonlocal means algorithm, that is, due to its huge amount of computational demand. The experimental scenarios we showed obtain great benefits by adopting a parallel approach using the GPU architecture and therefore encourage the exploration of more sophisticated algorithm variants and reduce the gap between the previous execution times and acceptable performance for real-time scenarios.

## Figures and Tables

**Algorithm 1:**
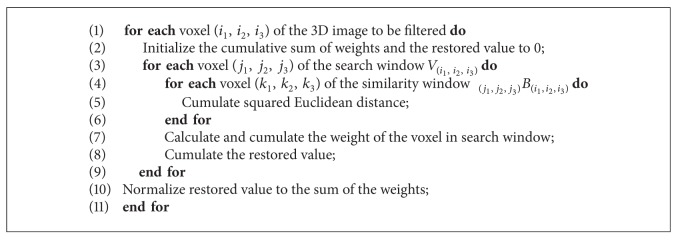
Pseudocode of the NLM algorithm.

**Algorithm 2:**
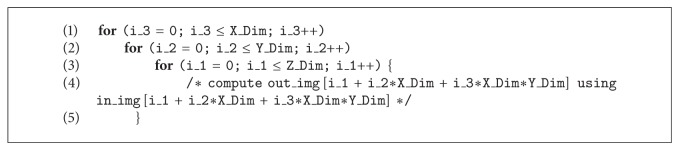
C code of NLM algorithm.

**Algorithm 3:**
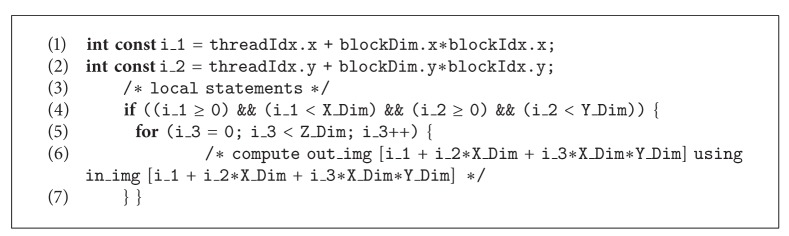
CUDA code of NLM algorithm with partial unrolling strategy.

**Algorithm 4:**
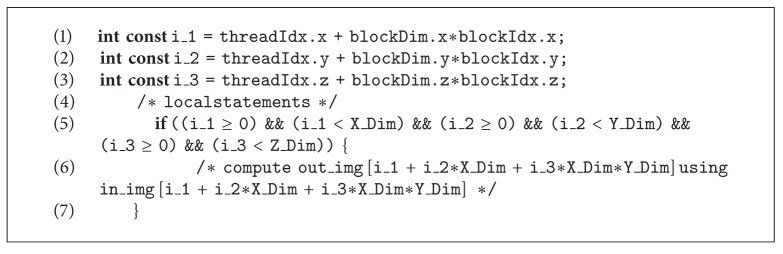
CUDA code of NLM algorithm with full unrolling strategy.

**Algorithm 5:**
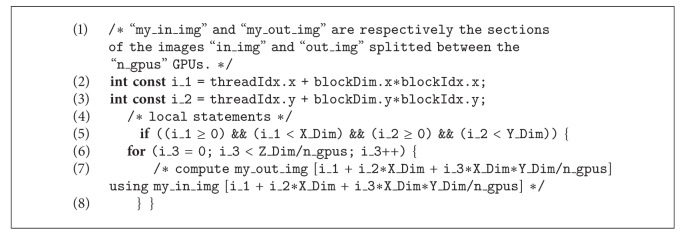
CUDA multi-GPU code of NLM algorithm with partial unrolling strategy.

**Table 1:** L1-prefer switch influence on execution times for the partial and full unrolling algorithm with (128, 1, 1) block size configuration and 3D datasets of normally distributed random numbers.

	Partial unrolling algorithm				Full unrolling algorithm			
	Single GPU		Multi-GPU		Single GPU		Multi-GPU	
	L1	no L1	L1	no L1	L1	no L1	L1	no L1
64^3^	0.77	0.83	0.38	0.42	0.58	0.58	0.23	0.23
128^2^ × 64	2.25	2.3	1.12	1.15	2.29	2.29	0.85	0.85
128^3^	4.5	4.59	2.25	2.3	4.38	4.38	1.9	1.9
256^2^ × 128	16.24	16.54	8.12	8.25	17.51	17.51	7.58	7.58
256^3^	32.5	33.58	16.24	16.54	34.22	34.24	15.94	15.95
512^2^ × 128	63.55	66.42	31.67	32.52	70.01	70	30.31	30.32
512^2^ × 256	128.19	138.06	63.57	66.59	136.89	136.95	63.75	63.77
512^3^	260.93	288.84	128.63	138.2	270.61	270.75	130.78	130.67

**Table 2:** Partial unrolling algorithm on a single GPU unit. Execution times and speed-up values for several block size configurations and 3D datasets of normally distributed random numbers. Search and similarity windows have been set according to |*V*_*i*_| = 11^3^ and |_*j*_ *B*_*i*_| = 3^3^.

Dataset size	Execution time/speed-up				
	1 GPU unit				CPU
	(16, 16, 1)	(128, 1, 1)	(256, 1, 1)	(512, 1, 1)	
64^3^	0.99/**17.7**	0.77/**22.7**	1.32/**13.3**	2.72/**6.43**	17.5
128^2^ × 64	2.31/**35.9**	2.25/**36.9**	2.23/**37.2**	4.57/**18.2**	83
128^3^	4.62/**36.8**	4.5/**37.8**	4.46/**38.1**	9.22/**18.4**	170
256^2^ × 128	16.9/**41.2**	16.2/**42.9**	16.6/**42**	18.6/**37.3**	696
256^3^	33.79/**41.2**	32.5/**42.9**	33.14/**42**	37.5/**37.2**	1393
512^2^ × 128	65.2/**43.1**	63.6/**44.3**	63.9/**44**	69.2/**40.7**	2814
512^2^ × 256	131/**43.1**	128/**43.9**	128/**43.8**	139/**40.5**	5623
512^3^	264/**42.7**	261/**43.3**	259/**43.5**	281/**40.2**	11291

**Table 3:** Partial unrolling algorithm on 2 GPU units. Execution times and speed-up values for several block size configurations and 3D datasets of normally distributed random numbers. Search and similarity windows have been set according to |*V*_*i*_| = 11^3^ and |_*j*_ *B*_*i*_| = 3^3^.

Dataset size	Execution time/speed-up				
	2 GPU units				CPU
	(16, 16, 1)	(128, 1, 1)	(256, 1, 1)	(512, 1, 1)	
64^3^	0.49/**35.7**	0.38/**46.1**	0.66/**26.5**	1.36/**12.9**	17.5
128^2^ × 64	1.15/**72.2**	1.12/**74.1**	1.11/**74.8**	2.27/**36.6**	83
128^3^	2.31/**73.6**	2.25/**75.6**	2.23/**76.2**	4.57/**37.2**	170
256^2^ × 128	8.44/**82.5**	8.12/**85.7**	8.28/**84.1**	9.26/**75.2**	696
256^3^	16.9/**82.5**	16.2/**85.8**	16.6/**84.1**	18.6/**74.7**	1393
512^2^ × 128	32.6/**86.2**	31.7/**88.9**	31.92/**88.2**	34.5/**81.3**	2814
512^2^ × 256	65.2/**86.2**	63.6/**88.5**	63.9/**88**	69.2/**81.3**	5623
512^3^	131/**86.5**	129/**87.8**	128/**87.9**	139/**81.2**	11291

**Table 4:** Full unrolling algorithm on a single GPU unit. Execution times and speed-up values for several block size configurations and 3D datasets of normally distributed random numbers. Search and similarity windows have been set according to |*V*_*i*_| = 11^3^ and |_*j*_ *B*_*i*_| = 3^3^.

Dataset size	Execution time/speed-up				
	1 GPU unit				CPU
	(16, 16, 1)	(128, 1, 1)	(256, 1, 1)	(512, 1, 1)	
64^3^	0.59/**29.7**	0.58/**30.2**	1.15/**15.2**	2.4/**7.29**	17.5
128^2^ × 64	2.33/**35.6**	2.29/**36.2**	2.3/**36.1**	4.8/**17.3**	83
128^3^	4.46/**38.1**	4.5/**37.8**	4.38/**38.8**	9.2/**18.5**	170
256^2^ × 128	17.9/**39**	17.5/**39.7**	17.5/**39.7**	18.4/**37.8**	696
256^3^	34.9/**39.9**	34.2/**40.7**	34.3/**40.6**	36/**38.7**	1393
512^2^ × 128	71.4/**39.4**	70/**40.2**	70.1/**40.1**	73.9/**38.1**	2814
512^2^ × 256	140/**40.3**	137/**41.1**	137/**41**	145/**38.9**	5623
512^3^	276/**40.9**	271/**41.7**	271/**41.7**	286/**39.5**	11291

**Table 5:** Full unrolling algorithm on 2 GPU units. Execution times and speed-up values for several block size configurations and 3D datasets of normally distributed random numbers. Search and similarity windows have been set according to |*V*_*i*_| = 11^3^ and |_*j*_ *B*_*i*_| = 3^3^.

Dataset size	Execution time/speed-up				
	2 GPU units				CPU
	(16, 16, 1)	(128, 1, 1)	(256, 1, 1)	(512, 1, 1)	
64^3^	0.12/**146**	0.23/**76.1**	0.43/**40.7**	0.9/**19.4**	17.5
128^2^ × 64	0.87/**95.4**	0.85/**97.6**	0.85/**97.6**	1.79/**46.4**	83
128^3^	1.94/**87.6**	1.9/**89.5**	1.9/**89.5**	3.99/**42.6**	170
256^2^ × 128	7.73/**90**	7.58/**91.8**	7.59/**91.7**	7.98/**87.2**	696
256^3^	16.3/**85.7**	15.9/**87.4**	16/**87.3**	16.8/**83**	1393
512^2^ × 128	30.9/**91.1**	30.3/**92.8**	30.3/**92.7**	32/**87.9**	2814
512^2^ × 256	65/**86.5**	63.8/**88.2**	63.8/**88.1**	67.3/**83.5**	5623
512^3^	133/**84.8**	131/**86.4**	131/**86.3**	138/**81.8**	11291

**Table 6:** Full unrolling algorithm on a single GPU unit. Execution times and speed-up values for a 3D dataset of normally distributed random numbers (size = 512 × 512 × 128) for several window configurations.

(|*V*_*i*_|, |_j_ *B*_*i*_|)	Execution time/speed-up				
	1 GPU unit				CPU
	(16, 16, 1)	(128, 1, 1)	(256, 1, 1)	(512, 1, 1)	
(11^3^, 3^3^)	71.4/**39.4**	70/**40.2**	70.1/**40.1**	73.9/**38.1**	2814
(21^3^, 3^3^)	514/**38.5**	504/**39.2**	505/**39.2**	538/**36.8**	19790
(11^3^, 5^3^)	254/**32**	244/**33.3**	244/**33.3**	267/**30.4**	8133
(21^3^, 5^3^)	1845/**31.9**	1761/**33.4**	1765/**33.3**	1964/**29.9**	58785

**Table 7:** Full unrolling algorithm on 2 GPU units. Execution times and speed-up values for a 3D dataset of normally distributed random numbers (size = 512 × 512 × 128) for several window configurations.

(|*V*_*i*_|, |_j_ *B*_*i*_|)	Execution time/speed-up				
	2 GPU units				CPU
	(16, 16, 1)	(128, 1, 1)	(256, 1, 1)	(512, 1, 1)	
(11^3^, 3^3^)	30.9/**91.1**	30.3/**92.8**	30.3/**92.7**	32/**87.9**	2814
(21^3^, 3^3^)	196/**101**	192/**103**	193/**103**	205/**96.5**	19790
(11^3^, 5^3^)	107/**75.8**	103/**78.9**	103/**78.8**	113/**72**	8133
(21^3^, 5^3^)	685/**85.8**	654/**89.8**	656/**89.7**	729/**80.6**	58785

**Table 8:** Partial unrolling algorithms for different video formats. Observe that the execution times include the time needed to copy data from CPU to GPU and from GPU to CPU.

Video size	MB	Execution time			
		(11^3^, 3^3^)	(21^3^, 3^3^)	(11^3^, 5^3^)	(21^3^, 5^3^)
PAL × 50	79.1	20.8	145	72.9	508
PAL × 100	158	41.6	291	146	1021
PAL × 200	316	83.7	586	294	2045
PAL × 500	791	212	1500	744	5162
HD × 50	148	39	271	136	953
HD × 100	297	78.1	545	274	1914
HD × 200	593	157	1104	551	3848
HD × 500	1483	398	2812	1395	9678
FULL-HD × 50	396	97.3	678	342	2386
FULL-HD × 100	791	195	1365	686	4788
FULL-HD × 200	1582	392	2754	1375	9601
FULL-HD × 500	3955	995	7018	3483	24149

**Table 9:** Full unrolling algorithms for different video formats. Observe that the execution times include the time needed to copy data from CPU to GPU and from GPU to CPU.

Video size	MB	Execution time			
		(11^3^, 3^3^)	(21^3^, 3^3^)	(11^3^, 5^3^)	(21^3^, 5^3^)
PAL × 50	79.1	16.8	85.8	55	276
PAL × 100	158	38.8	239	131	807
PAL × 200	316	82.9	545	284	1868
PAL × 500	791	215	1463	742	5052
HD × 50	148	31.4	161	103	517
HD × 100	297	72.7	448	246	1523
HD × 200	593	155	1022	532	3502
HD × 500	1483	403	2743	1391	9472
FULL-HD × 50	396	78.5	402	257	1294
FULL-HD × 100	791	182	1119	615	3781
FULL-HD × 200	1582	388	2550	1327	8738
FULL-HD × 500	3955	1007	6846	3473	23634

**Table 10:** Full unrolling algorithm with split for different video formats. Observe that the number of splits is intended on the same GPU and the time needed to copy data from CPU to GPU and from GPU to CPU is reported in bold.

Video size	Splits	Execution time + CPU-GPU copy time			
		(11^3^, 3^3^)	(21^3^, 3^3^)	(11^3^, 5^3^)	(21^3^, 5^3^)
PAL × 50	1	16.8 + **0.1**	85.8 + **0.15**	55 + **0.11**	276 + **0.16**
PAL × 100	2	33.5 + **0.2**	172 + **0.3**	110 + **0.22**	552 + **0.32**
PAL × 200	4	67 + **0.4**	343 + **0.59**	220 + **0.43**	1104 + **0.63**
PAL × 500	10	168 + **0.99**	858 + **1.48**	550 + **1.08**	2761 + **1.58**
HD × 50	1	31.4 + **0.18**	161 + **0.27**	103 + **0.2**	517 + **0.29**
HD × 100	2	62.8 + **0.37**	321 + **0.54**	206 + **0.4**	1035 + **0.58**
HD × 200	4	126 + **0.73**	643 + **1.09**	412 + **0.8**	2070 + **1.16**
HD × 500	10	314 + **1.83**	1607 + **2**	1030 + **4.8**	5175 + **2.9**
FULL-HD × 50	1	78.5 + **0.61**	402 + **1.04**	257 + **0.64**	1294 + **1.1**
FULL-HD × 100	2	157 + **1.23**	804 + **2.07**	515 + **1.28**	2587 +**2.2**
FULL-HD × 200	4	314 + **2.45**	1607 + **4.14**	1030 + **2.56**	5174 + **4.4**
FULL-HD × 500	10	785 + **6.14**	4018 + **10.4**	2575 + **6.4**	12935 + **11**
